# Establishing a reference interval for serum anti-dsDNA antibody: A large Chinese Han population-based multi-center study

**DOI:** 10.1371/journal.pone.0168871

**Published:** 2017-02-02

**Authors:** Chuiwen Deng, Shulan Zhang, Chaojun Hu, Ping Li, Ziyan Wu, Si Chen, Jing Li, Liubing Li, Fengchun Zhang, Yongzhe Li

**Affiliations:** 1 Department of Rheumatology and Clinical Immunology, Peking Union Medical College Hospital, Chinese Academy of Medical Sciences & Peking Union Medical College, Beijing, China; 2 Key Laboratory of Rheumatology and Clinical Immunology, Ministry of Education, Beijing, China; Peking University First Hospital, CHINA

## Abstract

**Background:**

A reference interval (RI) for the circulating concentration of anti-dsDNA antibody is essential for clinicians to interpret laboratory results and make clinical decisions. Therefore, we aimed to establish the RI for anti-dsDNA antibody in the Chinese Han population.

**Methods:**

This study was designed and carried out in accordance with guideline C28-A3, which is proposed by the International Federation of Clinical Chemistry and the Clinical and Laboratory Standards Institute. A total of 2,880 apparently healthy individuals were enrolled using *a posteriori* sampling. These individuals were recruited from four hospitals, representing the Han populations of north, south, east, and west China. Serum anti-dsDNA antibody levels were measured using the three analytical systems AESKU, EUROIMMUNE, and INOVA, which are the most commonly used systems in China. Individuals were stratified by gender, age, and region, and the RIs were obtained by nonparametric methods.

**Results:**

Gender-specific RIs for serum anti-dsDNA antibody in the Chinese Han population were established.

**Conclusion:**

This is the first exploration of the RI for anti-dsDNA antibody in the Chinese Han population. We have established gender-specific RIs for each assay method commonly used in China.

## Introduction

In laboratory medicine, reference intervals (RIs) represent the typical fluctuations in the quantity or quality of body fluid analytes in a relatively healthy population. The concept of an RI was first proposed by Grasbeck et al. in 1968 [1], and it was initially called a “normal value”. Later, it was realized that the term “normal” was scientifically flawed. Then, well-defined nomenclatures, including “reference value,” “reference range,” and “normal reference range” came into use. From a statistical standpoint, the term “reference interval” better fits the concept. Sometimes, an RI is confused with a clinical decision limit (CDL). A CDL is the threshold concentration of a body fluid analyte, and a specific medical decision is made when the concentration of an analyte for a given individual is above or below the CDL. Unlike an RI, a CDL is obtained from clinical studies that explore the diagnosis or specific outcome of a certain disease [2].

Generally, the manufacturers of diagnostic kits are obliged to provide the appropriate RI for clinical laboratories. In diagnostic kits for autoantibodies, most manufacturers provide cut-off values, which are used as RIs. However, not all RIs are rigorously calculated. One of the major issues in the application of RIs has been the lack of standardization in the selection of reference subjects. To address this problem, a standard protocol for establishing an RI (C28-A3) has been proposed by the International Federation of Clinical Chemistry together with the Clinical and Laboratory Standards Institute [3], and this has been widely used. In addition, the RIs provided with kits are typically calculated using reference subjects from the manufacturer’s country or region, and they are not necessarily applicable to individuals in other countries or regions. In China, most of the kits for autoantibody detection, which are procured from outside China, do not provide RIs based on Chinese or Asian populations, resulting in difficulties when evaluating RIs in clinical laboratories.

Fifty years ago, researchers found that circulating anti-dsDNA antibodies were present in patients with systemic lupus erythematosus (SLE) [4]. Subsequently, anti-dsDNA antibodies were shown to play important roles in SLE, both in its pathogenesis and as a biomarker for diagnosis and prognosis [5]. Thus, anti-dsDNA antibodies were introduced as a diagnostic biomarker in the classification and/or diagnostic criteria for SLE in 1982, 1997, and 2011 [6]. Then, a proposal was made that the criterion for the inclusion of anti-dsDNA antibody in the classification of SLE should be modified. It was suggested that the anti-dsDNA antibody level should be above the laboratory RI or twice the RI when tested by enzyme-linked immunosorbent assay [7]. Thus, calculating an accurate RI for the anti-dsDNA antibody level is important for making clinical decisions in SLE. Notably, there is a high incidence of SLE in China [8, 9], which makes it even more important to define an accurate RI for anti-dsDNA antibody in China.

To our knowledge, no study has explored the RI for anti-dsDNA antibody testing in a Chinese population. In the present study, we aimed to recruit a large number of apparently healthy Chinese Han individuals and establish RIs for anti-dsDNA antibody according to the standardized protocol.

## Methods

### Selection of reference subjects

Since the characteristics of autoantibodies have been poorly studied, and the current literature contains little relevant information, the factors that influence autoantibodies are little known. Based on this background, we chose *a posteriori* sampling, which was recommended by C28-A3 and fits the goal of our research. *A posteriori* sampling proceeds with the exclusion and partitioning of participants after sampling and analyte testing.

Reference subjects were selected from a population using specific, well-defined criteria, as recommended by C28-A3. In our study, we applied the following exclusion criteria: (1) a diagnosis of autoimmune disease (SLE, etc.); (2) a diagnosis of a disease that can affect immunoglobulin levels (liver cirrhosis, etc.); (3) activities or diseases that transiently affect the immune system, such as excessive smoking (more than 20 cigarettes per day), having one alcoholic drink every day for at least 2 weeks, surgery, or taking drugs that affect the immune system (non-steroidal anti-inflammatory drugs, disease-modifying anti-rheumatic drugs, immunosuppressors, glucocorticoids, gout suppressants, and biological agents), recent (prior few months) blood transfusion or blood donation, pregnancy, obesity (body mass index ≥ 28), confirmed malignancy, or diabetes. Since no RI for anti-nuclear antibody has been set, we could not include the status of this autoantibody as a criterion for eligibility. Written informed consent was not obtained for the essence of the study design. The utilized serum samples in the research were taken from the leftover samples of the routine tests, which will not influence the health or treatment of the participant. In this situation, verbal informed consent was obtained from all subjects and this was recorded by the physician who explained the merits of the project and the study procedure. The Ethics Committee of Peking Union Medical College Hospital approved this study, including the consent procedure.

According to the standard protocol for establishing an RI (C28-A3), to derive a 95% RI with the minimum subgroup of participants, 120 reference values, one from each reference subject is required [3]. Crucial data were recorded, including age, sex, and ethnic background. The information that authors had access to could not identify individual participants during or after data collection. A physical examination and certain clinical laboratory tests were performed as needed to assess the exclusion criteria and to confirm that the recruited participants were healthy individuals.

From January 2012 to Jun 2014, apparently healthy adults of the Han population were recruited from four hospitals in China, Peking Union Medical College Hospital, Guangdong General Hospital, Shanghai Changzheng Hospital, and West China Hospital, representing the Han populations from north, south, east, and west China, respectively. Finally, reference values obtained from 2,880 apparently healthy adults were processed to calculate RIs for anti-dsDNA antibody.

### Sample collection

Twelve hours prior to sample collection, participants were required to maintain their usual daily habits and diet but to avoid drinking alcohol and smoking. Under a fasting condition in the morning, blood samples were collected from the cubital vein and dispensed into 5-mL pro-coagulation tubes with gel (Becton, Dickinson, and Company, Franklin Lakes, NJ, USA). To separate the serum, blood samples were centrifuged at 3,000 rpm for 5 min within 6 h of collection. Each serum sample was equally divided into 5 tubes and frozen at −80°C until use.

### Assessment of the anti-dsDNA antibody tests

Analyte detection and statistical analysis were conducted from July 2014 to March 2016. Anti-dsDNA antibody levels were tested with three kits that are commonly used in China and are produced by EUROIMMUNE (Lubeck, Germany), AESKU (Wendelsheim, Germany), and INOVA (San Diego, CA, USA). Briefly, serum samples were diluted in sample buffer and then diluted serum, standards, and negative and positive controls were added to the designated antigen-coated micro wells and incubated at room temperature. Between all incubation steps, the wells were washed three times with wash buffer. Corresponding wells were probed by adding the respective secondary antibody conjugates and incubated at room temperature. Then, substrate was added to the respective wells and incubated at room temperature. Finally, stop solution was added and the optical density of each well was read.

### Statistical analysis

According to the C28-A3 guideline, a normal Q-Q plot was used to assess the data distribution before the statistical analysis. Combining the consensus and Q-Q plot analysis data, the measured anti-dsDNA antibody levels showed a skewed and non-Gaussian distribution. Therefore, a nonparametric method was used to calculate the RIs as the lower and upper reference limits. These two reference limits are the 2.5th and 97.5th percentiles of the distribution for the reference population. In addition, we performed a bootstrap procedure with 1,000 replicates to determine 90% CIs for both the lower and upper limits of the RIs.

The anti-dsDNA antibody RIs were calculated by the following two steps: First, a preliminary RI was calculated using all the data and the outliers were removed. The Dixon method, which is recommended by the C28-A3 guideline, has become widely used for identifying outliers. The Dixon method involves the calculation of the ratio D/R, where D is the absolute difference between an extreme observation (large or small) and the next largest (or smallest) observation, and R is the range of all observations, including extremes. If the difference D is equal to or greater than one-third of the range R, the extreme observation is considered an outlier and should be deleted. After excluding outliers, supplementary participants were recruited and anti-dsDNA antibody levels were measured. Second, the data were placed into subclasses by gender, and the RIs for males and females were calculated. Then, a Wilcoxon rank sum test was used to identify whether there were significant differences between the RIs. If there were no significant differences (*p* > 0.05), the data from males and females were combined for the statistical analysis. If there was a significant difference (*p* < 0.05), gender-specific RIs were calculated. In addition, data were subclassified according to age (16–30, 31–40, 41–50, 51–60, 61–70, and > 71 years) and district, and then the RI of each subclass was calculated.

The raw data of each group were utilized to perform comparisons between groups by Wilcoxon rank sum test, and *p* < 0.05 indicates a significant difference.

## Results

### Study population

The ages of the recruited apparently healthy adults ranged from 16 to 99 years. The study sample was divided into groups by decade of age (i.e., 31–40, 41–50, and 51–60 years), except for the groups of 16–30 and > 71 years old, which were set as the youngest and oldest age groups, respectively. The average age of the study participants is shown in S1 Table. According to the consensus view in the field [3], the minimum subgroup at one center should include 120 individuals (60 males and 60 females, S2 Table). After *a posteriori* sampling, 2,880 individuals were enrolled from the four regions, with 1,440 males and 1,440 females. There were no significant differences in mean age among the four regions or between the male and female groups.

### RI of Chinese Han

**Fig 1:**
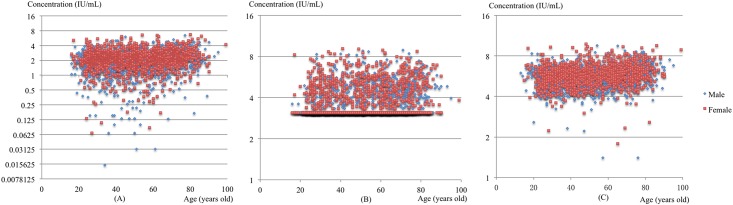
Scatter plots of the raw data for anti-dsDNA antibody. The logarithms of all raw data to base 2 were displayed on a scatter plot to visualize the data distribution for each of the three tested assay kits. (A) distribution of the data from AESKU; (B) distribution of the data from EUROIMMUNE; (C) distribution of the data from INOVA.

The logarithms of all raw data to base 2 are shown as scatterplots (Fig 1). The gender-specific RIs for anti-dsDNA antibody in the Chinese Han population are shown in Table 1. The Wilcoxon rank sum test results for every tested assay kit showed that there was a significant difference between the upper limits of the RIs for males and females (*p* < 0.05). Therefore, gender-specific RIs were calculated.

**Table 1:** Reference interval of anti-dsDNA antibody test in Han population of China.

Company	Units	Gender	2.5th (90% CI)	97.5th (90% CI)	Cut-off value recommended by kits
AESKU	IU/mL	Male	1.16 (1.10–1.27)	16.52 (15.02–17.78)	≥18IU/mL
		Female	1.45 (1.38–1.57)	22.06 (19.68–23.76)	
EUROIMMUNE	IU/mL	Male	8.60 (8.60–8.60)	103.17 (80.89–127.65)	≥100IU/mL
		Female	8.60 (8.60–8.60)	139.38 (123.36–161.74)	
INOVA	IU/mL	Male	15.37 (14.69–16.19)	182.92 (167.90–209.15)	≥200IU/mL
		Female	17.54 (16.78–18.69)	265.82 (224.60–286.71)	

### RIs for different age groups and districts

There was an apparent age-dependent variation in the RI. Generally, the older age groups, especially the group aged > 71 years, tended to have a higher upper limit of the RI (Table 2). The levels of anti-dsDNA antibody were compared between males and females of the same age group. Several age groups exhibited significantly higher levels of anti-dsDNA antibody in females than in males; in particular, the age groups 31–40 and 41–50 showed this result for each of the three tested assay kits.

**Table 2:** Reference intervals for anti-dsDNA antibody of different age groups of China.

Company	Units	Gender	Age group	2.5th (90% CI)	97.5th (90% CI)
AESKU	IU/mL	Male	16–30	1.11 (1.00–1.50)	14.40 (12.26–15.23)
			31–40	1.00 (1.00–1.11)	12.54 (10.38–16.50)
			41–50	1.05 (1.00–1.16)	12.59 (9.62–19.14)
			51–60	1.23 (1.14–1.39)	14.76 (12.27–19.55)
			61–70	1.43 (1.10–1.79)	15.45 (11.30–17.19)
			>71	1.45 (1.27–2.01)	25.49 (20.89–26.66)
		Female	16–30	1.22 (1.05–1.67)	22.31 (14.41–34.23)*
			31–40	1.45 (1.09–1.60)	14.79 (12.60–20.14)*
			41–50	1.47 (1.40–1.65)	22.79 (16.92–53.02)*
			51–60	1.23 (1.00–1.38)	17.70 (12.86–22.79)
			61–70	1.39 (1.00–1.68)	20.79 (17.19–36.06)*
			>71	1.87 (1.68–2.12)	31.60 (23.35–44.74)
EUROIMMUNE	IU/mL	Male	16–30	8.60 (8.60–8.60)	76.86 (37.01–127.83)
			31–40	8.60 (8.60–8.60)	76.35 (44.77–109.25)
			41–50	8.60 (8.60–8.60)	69.99 (62.38–158.28)
			51–60	8.60 (8.60–8.60)	150.61 (91.12–253.81)
			61–70	8.60 (8.60–8.60)	65.36 (48.80–125.22)
			>71	8.60 (8.60–8.60)	151.31 (112.70–233.80)
		Female	16–30	8.60 (8.60–8.60)	130.07 (75.75–187.52)*
			31–40	8.60 (8.60–8.60)	167.04 (82.00–322.48)*
			41–50	8.60 (8.60–8.60)	142.20 (124.07–271.57)*
			51–60	8.60 (8.60–8.60)	115.13 (80.23–163.38)
			61–70	8.60 (8.60–8.60)	159.23 (90.44–332.30)*
			>71	8.60 (8.60–8.60)	145.92 (115.86–202.21)
INOVA	IU/mL	Male	16–30	14.79 (11.85–15.51)	164.58 (114.52–266.47)
			31–40	14.66 (9.51–16.26)	132.42 (110.34–146.30)
			41–50	15.23 (12.23–18.30)	141.30 (109.10–208.57)
			51–60	14.41 (9.22–17.68)	266.77 (145.53–349.82)
			61–70	17.52 (14.70–20.95)	166.67 (130.76–239.62)
			>71	19.20 (13.43–22.05)	280.41 (208.44–365.83)
		Female	16–30	15.89 (12.68–17.46)	186.58 (134.98–299.78)
			31–40	17.28 (13.18–20.09)	134.61 (118.10–163.72)*
			41–50	16.98 (16.26–18.87)	392.34 (209.24–609.11)*
			51–60	17.65 (14.88–21.06)	218.67 (172.70–266.04)*
			61–70	20.83 (11.86–22.74)	314.09 (233.19–387.69)
			>71	19.99 (16.30–22.82)	316.56 (264.14–473.51)

* *p*<0.05, comparision of the levels of anti-dsDNA antibody between male and female in the same age group.

The RIs for the four centers in China were calculated (Table 3). The levels of anti-dsDNA antibody were compared between males and females at the same center. A few centers exhibited a significantly higher level of anti-dsDNA antibody in females than in males, especially the north and south centers, which showed this result for each of the three tested assay kits.

**Table 3:** Reference intervals for anti-dsDNA antibody of the four districts of China.

Company	Units	Gender	Region	2.5th (90% CI)	97.5th (90% CI)
AESKU	IU/mL	Male	North	1.76 (1.60–1.99)	18.08 (14.64–24.83)
			South	1.07 (1.00–1.16)	19.60 (17.14–27.86)
			East	1.09 (1.00–1.27)	10.00 (8.57–11.29)
			West	1.14 (1.02–1.26)	15.63 (12.61–17.21)
		Female	North	2.01 (1.84–2.22)	29.39 (20.08–42.23)*
			South	1.29 (1.01–1.60)	23.34 (19.36–40.88)*
			East	1.26 (1.06–1.45)	14.34 (10.58–22.55)*
			West	1.38 (1.22–1.61)	20.79 (14.64–26.35)*
EUROIMMUNE	IU/mL	Male	North	8.60 (8.60–8.60)	92.27 (64.56–128.68)
			South	8.60 (8.60–8.60)	121.76 (73.26–155.09)
			East	8.60 (8.60–8.60)	121.29 (66.67–192.61)
			West	8.60 (8.60–8.60)	112.63 (70.00–167.16)
		Female	North	8.60 (8.60–8.60)	185.40 (139.86–248.51)*
			South	8.60 (8.60–8.60)	169.02 (113.08–273.49)*
			East	8.60 (8.60–8.60)	107.75 (69.08–130.16)
			West	8.60 (8.60–8.60)	105.85 (67.36–137.53)
INOVA	IU/mL	Male	North	24.88 (20.27–26.74)	241.04 (194.85–282.50)
			South	20.97 (19.73–23.30)	143.22 (115.44–225.51)
			East	20.98 (6.25–26.07)	176.94 (166.73–194.53)
			West	12.23 (11.61–14.12)	142.25 (125.61–208.16)
		Female	North	23.06 (17.70–25.15)	296.62 (265.61–552.24)*
			South	19.37 (16.01–23.28)	235.26 (196.71–290.15)*
			East	26.39 (22.20–30.56)	285.67 (220.23–456.23)*
			West	14.03 (11.87–15.73)	175.34 (127.64–284.58)*

* *p*<0.05, comparision of the levels of anti-dsDNA antibody between male and female in the same center.

### Comparison of the calculated RI and provided cut-off values

Among the three kits, the methods of defining cut-off value were diverse. The EUROIMMUNE recruited 206 healthy blood donors and tested the levels of the anti-dsDNA antibodies. They set the cut-off value as 100 IU/ml for 1.5% of the blood donors were anti-dsDNA antibody positive when applying that value. In addition, INOVA declared that the normal range for their assay was determined by analyzing samples from 175 random blood donors. Of that number all but 1.1% had anti-dsDNA antibody values less than 200 IU/ml. The AESKU did not describe how the cut-off value was defined. All these cut-off values were not supposed to be the upper limit of a RI (i.e., the 97.5% of a reference population). In our study, the numerical values of the calculated gender-specific RIs and the cut-off values provided for the tested assay kits were diverse. Most of the cut-off values were not suitable for clinical use. Many of the 90% CIs that we calculated for the 97.5 percentile did not include the assay cut-off values, although some of them did.

### Identification and handling of outliers

According to the Dixon method [10], there were four outliers in the AESKU assay, and one in the EUROIMMUNE assay [10]. No outliers were observed in the INOVA assay. Therefore, five additional apparently healthy adults were added to replace the outliers (Table 4).

**Table 4:** The test results of the outliers and the substitutes.

Population	Manufacturer	Gender	Outliers		Substitutes	
			Age	Result	Age	Result
South of China	EUROIMMUNE	Male	65	>max	64	0
South of China	AESKU	Male	81	138.27	81	26.61
South of China	AESKU	Female	41	139.67	44	14.88
West of China	AESKU	Male	78	>max	78	8.19
West of China	AESKU	Female	69	>max	69	11.18

## Discussion

The RI is important for clinical decision-making, including diagnosis, evaluation of disease activity, and prognosis. Clinical decision-making for autoimmune diseases mainly relies on the detection of autoantibodies. Typically, the manufacturer of a test kit provides cut-off values, which are used as the RI in clinical practice. However, most of the kits currently used to measure autoantibodies in China are imported kits, and their cut-off values have been derived from foreign populations and have not been systematically calculated. Differences in ethnic background or race could lead to a disparity in the RIs for immunologic indices, for example, serum autoantibodies [11]. Although anti-dsDNA antibody is an important immunologic index for autoimmune disease, its RI has not been systematically defined until now. Here, we conducted this study to establish RIs for anti-dsDNA antibody in the Chinese Han population according to guideline C28-A3.

In this research, gender-specific RIs for anti-dsDNA antibody were observed in the Chinese Han population (Table 1). Generally, cut-off values are set at a point that is convenient for clinical use. In our study, the RIs for anti-dsDNA antibody were far higher or lower than the cut-off values provided by the manufacturers of the tested assay kits. Many of the 90% CIs that we calculated for the 97.5 percentile did not include the assay cut-off values, although some of them did. The differences between the provided cut-off values and our calculated RIs may be explained by two factors. First, the cut-off values were calculated using non-Chinese populations. Second, the methods for calculating cut-off values, including recruiting participants and statistical analysis, have not been standardized among kit manufacturers. Our study established accurate and detailed RIs for anti-dsDNA antibody testing according to the standard protocol, C28-A3, and these RIs could help clinicians to increase the accuracy of their clinical decisions.

It was interesting to find that, in the age groups of 31–40 and 41–50 years, the levels of anti-dsDNA antibody were higher in females than in males (Table 2). In addition, significantly higher levels of anti-dsDNA antibody were found in females recruited from the north and south centers than in males from the same centers (Table 3). These results suggest that unknown factors may influence anti-dsDNA antibody concentrations in the above-mentioned age groups or centers, and specific cut-off values should be set for them. In addition, we observed that the older age groups tended to have higher values of RIs, particularly the population aged 71 years and older. We speculate that changes occur with age in females that result in abnormal immune function, especially the production and clearance of autoantibodies.

Of note, the significant differences that we discussed above might not only be attributed to gender, age, and region, but could also result from random variance and others. Therefore, additional related studies should be performed to explore the pathogenesis of autoimmune disease and provide more information regarding other factors that influence the RI for anti-dsDNA antibody.

There were five outliers (Table 4), which were replaced with new participants. Since we ruled out participants with autoimmune diseases by a physical examination and clinical laboratory tests, the anti-dsDNA antibody levels of these outliers might be elevated for other physiological reasons, or the elevation might be indicative of the onset of related autoimmune diseases, as other autoantibodies, such as anti-Sm antibody, can be detected several years before disease onset. On the latter basis, these five outliers warrant long-term follow up.

In conclusion, this is the first study to have explored the RI for anti-dsDNA antibody in the Chinese Han population. Gender-specific RIs were established, and their application may help clinicians to make accurate decisions for Chinese Han individuals.

## Supporting information

S1 Table Average age of recruited participant from different centers of China. (DOC)

S2 Table The number of participant recruited from different centers of China. (DOC)

## References

[1] Gräsbeck R, Fellman J. Normal values and statistics. Scand J Clin Lab Invest. 1968;21:193–195. 5708691 10.3109/00365516809076984

[2] Sikaris KA. Physiology and its importance for reference intervals. Clin Biochem Rev. 2014;35:3–14. 24659833 PMC3961997

[3] Horowitz G, Altaie 1S, Boyd JC, Ceriotti F, Garg U, Horn P, et al. Defining, establishing, and verifying reference intervals in the clinical laboratory. Approved guideline, 3rd ed. Clinical Laboratory and Standards Institute; 2008.

[4] Holman HR, Kunkel HG. Affinity between the lupus erythematosus serum factor and cell nuclei and nucleoprotein. Science. 1957;126:162–163. 13442661 10.1126/science.126.3265.162

[5] Isenberg DA, Manson JJ, Ehrenstein MR, Rahman A. Fifty years of anti-ds DNA antibodies: are we approaching journey's end? Rheumatology (Oxford). 2007;46:1052–1056. 17500073 10.1093/rheumatology/kem112

[6] Fu SM, Dai C, Zhao Z, Gaskin F. Anti-dsDNA Antibodies are one of the many autoantibodies in systemic lupus erythematosus. F1000Res. 2015;4:939 10.12688/f1000research.6875.1 26594353 PMC4648223

[7] Petri M, Orbai AM, Alarcón GS, Gordon C, Merrill JT, Fortin PR, et al Derivation and validation of the Systemic Lupus International Collaborating Clinics classification criteria for systemic lupus erythematosus. Arthritis Rheum. 2012;64:2677–2686. 10.1002/art.34473 22553077 PMC3409311

[8] Zou YF, Feng CC, Zhu JM, Tao JH, Chen GM, et al Prevalence of systemic lupus erythematosus and risk factors in rural areas of Anhui Province. Rheumatol Int. 2014;34:347–356. 10.1007/s00296-013-2902-1 24264010

[9] Mok CC, To CH, Ho LY, Yu KL. Incidence and mortality of systemic lupus erythematosus in a southern Chinese population, 2000–2006. J Rheumatol. 2008;35:1978–1982. 18688913

[10] Dixon WJ. Processing data for outliers. Biometrics. 1953;9:74–89.

[11] Schauer U, Stemberg F, Rieger CH, Borte M, Schubert S, Riedel F, et al IgG subclass concentrations in certified reference material 470 and reference values for children and adults determined with the binding site reagents. Clin Chem. 2003;49:1924–1929. 14578325 10.1373/clinchem.2003.022350

