# Supplementary material for: Establishing a reference interval for serum anti-dsDNA antibody: A large Chinese Han population-based multi-center study
Source: PLoS One. 2017 Feb 2;12(2):e0168871. doi: 10.1371/journal.pone.0168871 (PMC5289441; doi:10.1371/journal.pone.0168871)
Supplement: S1 Table — (DOC) [file pone.0168871.s001.doc]

**S1 Table. Average age of recruited participant from different centers of China.**

| Population | Male |  | Female |
| --- | --- | --- | --- |
|  | Mean±SD |  | Mean±SD |
| East of China | 50.62±18.13 | | 51.01±18.15 |
| West of China | 50.39±17.34 | | 50.23±16.82 |
| South of China | 50.96±17.66 | | 50.69±17.66 |
| North of China | 50.49±17.56 | | 50.75±17.26 |
