# Supplementary material for: Establishing a reference interval for serum anti-dsDNA antibody: A large Chinese Han population-based multi-center study
Source: PLoS One. 2017 Feb 2;12(2):e0168871. doi: 10.1371/journal.pone.0168871 (PMC5289441; doi:10.1371/journal.pone.0168871)
Supplement: S2 Table — (DOC) [file pone.0168871.s002.doc]

**S2 Table. The number of participant recruited from different centers of China.**

|  | No.of the North | | |  | | No.of the East | | |  | No.of the South | | |  | No.of the West | | |
| --- | --- | --- | --- | --- | --- | --- | --- | --- | --- | --- | --- | --- | --- | --- | --- | --- |
|  | Male |  | Female | | | Male |  | Female |  | Male |  | Female |  | Male |  | Female |
| 16-30 | 60 |  | 60 | |  | 60 |  | 60 |  | 60 |  | 60 |  | 60 |  | 60 |
| 31-40 | 60 |  | 60 | |  | 60 |  | 60 |  | 60 |  | 60 |  | 60 |  | 60 |
| 41-50 | 60 |  | 60 | |  | 60 |  | 60 |  | 60 |  | 60 |  | 60 |  | 60 |
| 51-60 | 60 |  | 60 | |  | 60 |  | 60 |  | 60 |  | 60 |  | 60 |  | 60 |
| 61-70 | 60 |  | 60 | |  | 60 |  | 60 |  | 60 |  | 60 |  | 60 |  | 60 |
| >71 | 60 |  | 60 | |  | 60 |  | 60 |  | 60 |  | 60 |  | 60 |  | 60 |
